# La trisomie 18 ou syndrome d'Edwards en post-natal: étude descriptive au Centre Hospitalier Universitaire de Casablanca et revue de littérature

**DOI:** 10.11604/pamj.2020.37.309.26205

**Published:** 2020-12-03

**Authors:** Fatima Zahra Outtaleb, Rachida Errahli, Nora Imelloul, Ghizlane Jabrane, Nadia Serbati, Hind Dehbi

**Affiliations:** 1 Laboratoire de Génétique Médicale, Centre Hospitalier Universitaire Ibn Rochd de Casablanca, Casablanca, Maroc,; 2 Laboratoire de Pathologie Cellulaire et Moléculaire, Faculté de Médecine et de Pharmacie de Casablanca, Université Hassan II, Casablanca, Maroc

**Keywords:** Trisomie 18, dysmorphie faciale, diagnostic prénatal, Trisomy 18, facial dysmorphism, prenatal diagnosis

## Abstract

La trisomie 18 est une maladie chromosomique, dû à la présence d'un chromosome 18 surnuméraire. Les nourrissons atteints de trisomie 18 ont un taux de mortalité élevé, secondaire aux malformations létales associées à ce syndrome. L´objectif de cette étude est de décrire les caractéristiques cliniques et cytogénétiques de ces patients, ainsi que l´intérêt du conseil génétique. C´est une étude descriptive transversale réalisée sur une période de 5 ans, allant de juillet 2015 à avril 2019. L´étude a concerné les patients suivis au service de génétique médicale du Centre Hospitalier Universitaire (CHU) Ibn Rochd de Casablanca et présentant des anomalies évoquant la trisomie 18, et confirmé par étude cytogénétique. Il s´agit de 5 patients atteints du syndrome d´Edwards, suspectés cliniquement, puis confirmé à l´étude cytogénétique, avec une prédominance féminine; 3 filles et 2 garçons (sex-ratio = 0,67). L´âge moyen au moment du diagnostic était de 37,40 ± 23,98 jours (9 jours-2 mois). La trisomie 18 a été évoquée cliniquement dans deux cas devant une dysmorphie faciale et un syndrome malformatif caractéristiques de l´anomalie chromosomique, alors que deux patientes étaient hospitalisées en unité de soins intensifs pour insuffisance cardiaque décompensée, sur cardiopathie congénitale, et un patient a présenté une détresse respiratoire néonatale sur un syndrome poly malformatif, au moment du diagnostic. L´étude cytogénétique réalisée a confirmé le diagnostic de trisomie 18 libre et homogène chez les cinq patients, puis un conseil génétique a été réalisé. La prévalence de la trisomie 18 est variable. Au niveau mondial, on estime la prévalence 1/6000 naissances vivantes, les plus touchées étant celles du sexe féminin. Le diagnostic de trisomie 18 peut être suspecté à la naissance chez un nouveau-né présentant une dysmorphie crânio-faciale caractéristique, et une position du « suppliant » des bras, mains avec des doigts en flexion permanente, l´index chevauchant le 3^e^ doigt, l´auriculaire chevauchant le 4^e^ doigt. Il existe plusieurs malformations associées à la trisomie 18. Le syndrome est également évoqué en anténatal en cas de présence d´anomalies à l´échographie obstétricale. Par ailleurs, la survie est faible et seul un nouveau-né sur 10 atteint la première année de vie.

## Abstract (en)

Trisomy 18 is a chromosomal disease, caused by the presence of a supernumerary chromosome 18. Mortality among infants with trisomy 18 is high, secondary to lethal malformations associated with this syndrome. The purpose of this study was to describe the clinical and cytogenetic features of these patients, as well as the role of genetic counselling. We conducted a cross-sectional descriptive study over a 5-year period, from July 2015 to April 2019. The study involved, patients followed up in the Department of Medical Genetics at the University Hospital Center Ibn Rochd of Casablanca, having abnormalities suggestive of trisomy 18, then confirmed by cytogenetic study. The study enrolled 5 patients, 3 girls and 2 boys (female predominance; sex-ratio = 0,67) with clinically suspected Edward’s syndrome, then confirmed by cytogenetic study. The mean age at diagnosis was 37.40 ± 23.98 days (9 days-2 months). Trisomy 18 was clinically suspected in two cases based on facial dysmorphism and malformative syndrome, a recognizable pattern of chromosomal abnormality. Two patients were hospitalized in the intensive care unit for decompensated heart failure associated with congenital heart disease, while one patient had neonatal respiratory distress associated with polymalformative syndrome at diagnosis. Cytogenetic study confirmed the diagnosis of free and homogeneous trisomy 18 in five patients, then genetic counselling was performed. The prevalence of trisomy 18 is variable. Global prevalence is estimated at 1/6000 live births, females are mostly affected. The diagnosis of trisomy 18 should be suspected at birth in newborns with typical craniofacial dysmorphism, arms lifted in supplication and permanent flexion of the fingers, the index finger overlapping the 3^rd^ finger, the little finger overlapping the 4^th^ finger. There are several malformations associated with trisomy 18. This syndrome should be also suspected in the antenatal period in patients with abnormalities on obstetric ultrasound. Moreover, survival is low and only one in 10 newborns reach the first year of life.

## Introduction

La trisomie 18 est une maladie chromosomique constitutionnelle, définie par la présence d'un chromosome 18 surnuméraire. C´est la trisomie autosomique la plus fréquente après la trisomie 21, ou syndrome de Down [1]. Les nourrissons atteints de trisomie 18 ont un taux de mortalité élevé, secondaire aux malformations létales associées à ce syndrome. Seulement 4% peuvent survivre à leur première année de vie [2].

**Intérêts de l´étude:** décrire les caractéristiques cliniques et cytogénétiques de ces patients, ainsi que l´intérêt du conseil génétique. Intérêt du caryotype constitutionnel dans la prise en charge des nouveau-nés présentant une hypotrophie néonatale et/ou un retard de croissance intra-utérin (RCIU) harmonieux.

## Méthodes

C´est une étude descriptive transversale réalisée sur une période de 5 ans, allant de juillet 2015 à avril 2019. L´étude a concerné tous les patients suivis au service de génétique médicale du CHU Ibn Rochd de Casablanca, et présentant des anomalies évoquant la trisomie 18, qui a été confirmée par une étude cytogénétique. L´analyse du caryotype constitutionnel en bandes Reverse (résolution 400 bandes par lot haploïde) a été réalisée sur les lymphocytes d´un prélèvement de sang veineux périphérique sur tube hépariné, après mise en culture durant 72 heures dans un milieu composé de: RPMI 1640, sérum de veau fœtal, phyto-hémaglutinine, et antibiotiques (streptomycine et pénicilline). Par la suite, il a été réalisé un blocage de la culture cellulaire par la colchicine, puis un choc hypotonique par une solution hypotonique de chlorure de potassium, permettant la libération des chromosomes métaphasiques. Enfin, l´examen sous microscope optique a été réalisé, après la fixation, l´étalement des lames, la dénaturation thermique, et la coloration au May Grunwald Giemsa.

## Résultats

**Figure 1:**
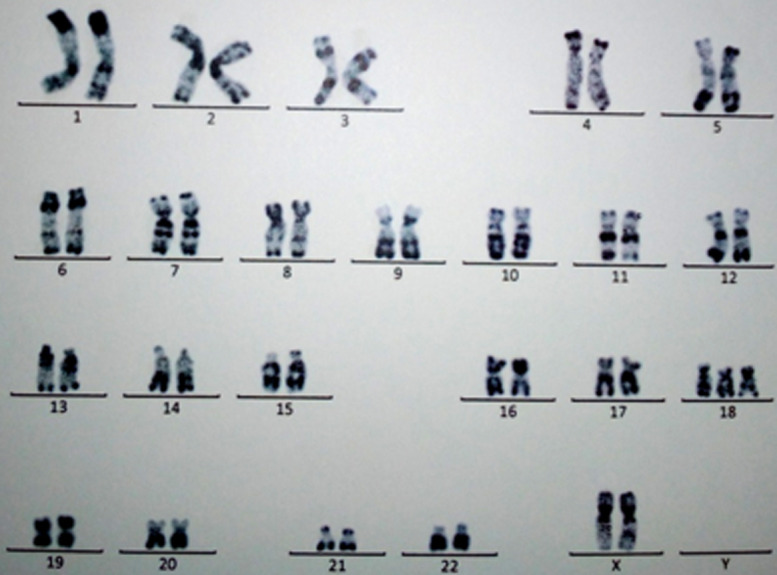
l’étude cytogénétique en bandes RHG, résolution 400 bandes, de la patiente FD, met en évidence un caryotype féminin, et la présence d’un chromosome 18 surnuméraire, confirmant le diagnostic de trisomie 18 libre et homogène: 47,XX+18

**Figure 2:**
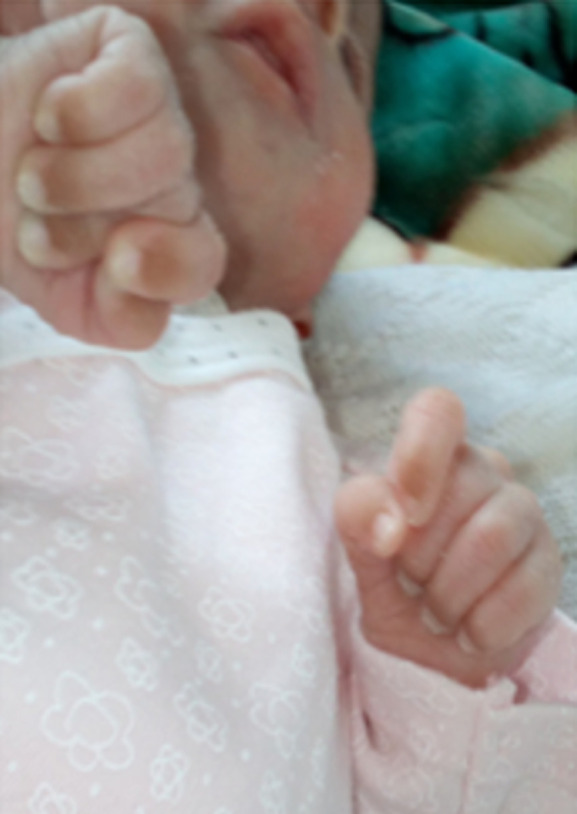
phénotype de la patiente C.E, à j58 de vie, montrant la dysmorphie faciale et les poings fermés, avec l’index recouvrant le troisième doigt au niveau des deux mains

Il s´agit de 5 patients atteints du syndrome d´Edwards, suspecté cliniquement, puis confirmé à l´étude cytogénétique. L´âge moyen au moment du diagnostic était de 37,40 ± 23,98 jours (9 jours-2 mois), avec une prédominance féminine: 3 filles et 2 garçons (sex-ratio = 0,67). Ces patients étaient adressés initialement en consultation de génétique médicale, devant la suspicion clinique de trisomie 18, révélée dans deux cas (patiente C.E et patient N.L) par une dysmorphie faciale et une hypotrophie néonatale (Figure 1, Figure 2), alors que deux patientes (C.S et F.D) étaient hospitalisées en unité de soins intensifs du service des maladies cardio-respiratoires de l´hôpital d´Enfant pour insuffisance cardiaque décompensée, sur cardiopathie congénitale. Enfin, le patient Y.M était admis au service de néonatologie pour détresse respiratoire néonatale sur syndrome poly malformatif. L´étude cytogénétique réalisée a permis la mise en évidence de la présence d´un chromosome surnuméraire pour la 18^e^ paire autosomique, confirmant ainsi le diagnostic de trisomie 18 dans sa forme libre et homogène chez les cinq patients. Le Tableau 1 résume les principales caractéristiques cliniques et cytogénétiques de ces patients.

**Tableau 1:** caractéristiques cliniques et cytogénétiques des patients atteints de trisomie 18, suivis au CHU de Casablanca entre juillet 2015 à avril 2019

Observations	Age au moment du diagnostic (en jours)	Sexe	Anomalies au cours de la grossesse	Dysmorphie faciale	Syndrome malformatif	Complications évolutives	Résultat de l'étude du caryotype constitutionnel
Patiente CE	58	Féminin	RCIU	-Dolichocéphalie	-Hypotrophie néonatale	-	47,XX+18
				-Microrétrognatisme			
				-Petite bouche	-Poings fermés, avec index recouvrant le troisième doigt		
				-Oreilles bas implantées, mal ourlées			
Patient YM	9	Masculin	RCIU	-Occiput saillant	-Chevauchement des doigts de la main	Détresse respiratoire néonatale	47,XY+18
				-Oreilles bas implantées			
					-Hypogénitalisme		
Patient NL	15	Masculin	-	-	-Hypotrophie néonatale	-	47,XY+18
					-Syndrome polymalformatif		
Patiente CS	45	Féminin	-	-	Syndrome polymalformatif	Insuffisance cardiaque décompensée sur cardiopathie congénitale	47,XX+18
Patiente FD	60	Féminin	RCIU	Dysmorphie faciale	- Hypotrophie	Insuffisance cardiaque décompensée sur cardiopathie congénitale	47,XX+18
					- Luxation congénitale des hanches		
					-Plusieurs déformations squelettiques		

## Discussion

La prévalence de la trisomie 18 est variable. Au niveau mondial, elle est estimée à 1/6000 naissances vivantes, les plus touchées étant celles du sexe féminin [3]. En post-natal, le diagnostic du syndrome d´Edwards est évoqué cliniquement devant un nouveau-né hypertonique avec un syndrome poly-malformatif. Comme la plupart des pathologies chromosomiques autosomiques, il s´accompagne aussi d´un retard de croissance (trisomie 21 pour la plus connue) [4]. La dysmorphie crânio-faciale, caractéristique de ce syndrome associe une microcéphalie avec saillie de l´occiput, un front fuyant, des oreilles bas implantées et pointues caractéristiques, une bouche petite avec un palais ogival et une micro-rétrognathie. Les anomalies des extrémités sont représentées par la position du « suppliant » des avant-bras, les poings fermés mains et les doigts en flexion permanente. Ainsi, l´index chevauche le 3^e^ doigt et l´auriculaire chevauche le 4^e^ doigt. Le bassin est étroit avec des pieds bots varus équins et en piolet.

Il existe plusieurs malformations viscérales associées à la trisomie 18, notamment cardiaques, pulmonaires, rénales et digestives, avec omphalocèle et hernies diaphragmatiques [5]. Les cardiopathies congénitales ont été retrouvées chez deux patientes dans cette étude, et les anomalies squelettiques chez un seul cas. En salle de travail, la seule urgence thérapeutique caractéristique est l´aide technique rationnelle devant une malformation manifestée par une détresse vitale [6]. Par ailleurs, la survie est faible et seul un nouveau-né sur 10 atteint la première année de vie, les nourrissons de sexe féminin ayant la durée de survie la plus longue [3]. Les principales causes de décès sont les myocardiopathies, l'insuffisance cardiaque et l´insuffisance respiratoire [7-9]. Ainsi, la survie est de 42% la première semaine; 29% au premier mois, 12% à 3 mois et 8% à 6 mois [2].

**Conseil génétique:** un conseil génétique doit être réalisé, dans lequel il est expliqué que pour un couple avec un enfant atteint de trisomie 18 libre et homogène, la probabilité de récurrence lors de la prochaine grossesse est de 1% [3, 8]. Dans les cas où la trisomie 18 est partielle, il est nécessaire d'effectuer un caryotype chez les parents pour éliminer les porteurs avec une translocation équilibrée, incluant le segment trisomique, car dans ces cas, la probabilité de récurrence est plus grande [3]. Par ailleurs, l´incidence de la trisomie 18 augmente en cas d´âge maternel avancé. Quatre-vingts pourcent des cas sont le résultat de la non-disjonction méiotique maternelle, et 5% des cas sont dus à la non-disjonction méiotique paternelle. Exceptionnellement, la trisomie 18 est secondaire à une translocation chromosomique [1].

**Diagnostic prénatal:** la complexité et la sévérité du tableau clinique à la naissance et le taux élevé de mortalité néonatale et infantile soulignent l´intérêt du diagnostic prénatal de cette pathologie. En pathologie chromosomique, en cas d´antécédent d´enfant avec une anomalie chromosomique ou la présence chez l´un des parents d´un remaniement chromosomique, un caryotype fœtal est proposé à la recherche d´une anomalie chromosomique chez le fœtus. En l´absence de ces antécédents, la trisomie 18 est évoquée en anténatal grâce à l´échographie obstétricale morphologique. Ainsi, les principaux signes d´appels échographiques sont le retard de croissance intra-utérin, l'augmentation de la clarté nucale et l'absence d'os nasal (également utilisés dans le syndrome de Down et le syndrome de Patau) qui sont observées chez 66% des fœtus atteints de trisomie 18 [10], ainsi que les signes en faveur de malformations viscérales et des extrémités, notamment les poings fermés de façon permanente. Dans son approche conventionnelle, le diagnostic prénatal d´une maladie génétique, chromosomique, comme la trisomie 18, repose sur les analyses cytogénétiques de prélèvements d´origine fœtale, obtenue par les actes invasifs que sont la choriocentèse (villosités choriales), l´amniocentèse (liquide amniotique) ou la cordocentèse (sang fœtal). Ces analyses réalisées relèvent de la cytogénétique conventionnelle (caryotype) ou moléculaire (hybridation in situ FISH) [11]. Les outils de la biologie moléculaire peuvent permettre également d´accéder à l´information sur le nombre de chromosomes 18 par analyse de l´ADN par l´analyse de polymorphismes génétiques ou par des techniques de quantification relative du génome [11].

## Conclusion

Cette étude descriptive souligne l´intérêt du caryotype constitutionnel dans la prise en charge des nouveau-nés présentant une hypotrophie néonatale et/ou un RCIU harmonieux, surtout en l´absence de diagnostic anténatal. La trisomie 18, étant une pathologie de mauvais pronostic, la prise en charge se limite aux soins de confort, vu que la prise en charge chirurgicale des malformations viscérales associées n´améliore pas le pronostic [2].

### Etat des connaissances sur le sujet

- La trisomie 18 en post-natal est une pathologie rare, de mauvais pronostic;
- L´analyse cytogénétique est essentielle pour la confirmation diagnostic.

### Contribution de notre étude à la connaissance

- A notre connaissance, l´analyse descriptive de cette maladie rare, rapportée dans cette étude est parmi les premières séries publiées au niveau national.
